# G-CSF Infusion for Stem Cell Mobilization Transiently Increases Serum Cell-Free DNA and Protease Concentrations

**DOI:** 10.3389/fmed.2020.00155

**Published:** 2020-04-28

**Authors:** Maria Stoikou, Shane V. van Breda, Günther Schäfer, Lenka Vokalova, Stavros Giaglis, Alexandra Plattner, Laura Infanti, Andreas Holbro, Sinuhe Hahn, Simona W. Rossi, Andreas Buser

**Affiliations:** ^1^Department of Biomedicine, University of Basel and University Hospital of Basel, Basel, Switzerland; ^2^Blood Transfusion Service, Swiss Red Cross Basel, Basel, Switzerland

**Keywords:** G-CSF, stem cell mobilization, PMN, MPO, NE, ROS, cell-free DNA, NETs

## Abstract

G-CSF for stem cell mobilization increases circulating levels of myeloid cells at different stages of maturation. Polymorphonuclear cells (PMNs) are also mobilized in high numbers. It was previously reported that G-CSF primes PMNs toward the release of neutrophils extracellular traps (NETs). Since NETs are often involved in thrombotic events, we hypothesized that high G-CSF blood concentrations could enhance PMN priming toward NET formation in healthy hematopoietic stem cell donors, predisposing them to thrombotic events. However, we found that G-CSF does not prime PMNs toward NETs formation, but increases the serum concentration of cell-free DNA, proteases like neutrophils elastase and myeloperoxidase, and reactive oxygen species. This could possibly create an environment disposed to induce thrombotic events in the presence of additional predisposing factors.

## Introduction

Granulocyte colony-stimulating factor (G-CSF) is playing an essential role as a colony-stimulating factor during hematopoiesis. G-CSF stimulates proliferation and differentiation of hematopoietic stem cells (HSC) (1), differentiation of precursor cells into mature PMNs, and the release of mature granulocytes and hematopoietic stem cells from the bone marrow (2). The latter has led to the widespread use of G-CSF (3) for the mobilization of HSC for transplant purposes (4, 5). Findings from the last 20 years indicate that HSC mobilization with G-CSF is associated with an increased hypercoagulable state and therefore a higher risk for thrombotic events (6).

PMNs play a leading role in innate immunity (7); they are rapidly recruited to sites of inflammation where they phagocytose, degranulate, or generate NETs (8). The release of NETs is an active and rapid process that leads to a specific form of PMN cell death, called NETosis (9). NETs are composed of nuclear DNA decorated with histones and granular proteins such as neutrophil elastase (NE) and myeloperoxidase (MPO). One key regulator of NET formation in the citrullination of Histone H3 mediated by the enzyme PAD4. It was described that NETs can promote thrombosis (10) since histones H3 and H4 enhance platelet recruitment by activation of factor XII (11). Moreover, NE, MPO and reactive oxygen species (ROS) seem to regulate the clotting cascade and promote coagulation by cleavage and oxidation of anticoagulants; tissue factor pathway inhibitor (TFPI) and thrombomodulin (TM) (12). Recently it was observed that increased levels of circulatory G-CSF contributed to the regulation of NETosis during pregnancy (13), which is also associated with increased risk for thrombosis.

The goal of our study was to analyse the occurrence and dynamics of NET formation in healthy stem cell donors at the time of stem cell collection and follow-up.

We observed that circulating PMNs mobilized after G-CSF application present markers of immaturity, correlate with high cell-free DNA and increased protease content. This effect was of short duration, with all parameters returning back to baseline 1-month post-G-CSF infusion.

## Materials and Methods

### Donors

Allogeneic peripheral stem cell donors were asked to participate in the study. Donor eligibility was assessed according to internal policies and in compliance with the current FACT-JACIE standards (https://www.ebmt.org/jacie-standards). G-CSF (Neupogen^®^, Amgen) was applied at a dose of 10 μg/kg body weight divided into two doses daily (total: eight injections) subcutaneously, beginning 5 days before HSC harvest.

This study was approved by the ethical committee of Northern and Central Switzerland (2015-00191).

### Samples Collection and PMN Isolation

Samples were collected from HSC donors and healthy age-matched controls at various time points; 3–4 weeks prior to G-CSF administration (visit A); at the time of HSC harvesting (visit B); 1 and 3 months post apheresis (visit C and D).

Whole blood was collected into EDTA- and silicone-coated tubes (Sarstedt), and analyzed by a Advia (Siemens) for complete blood cell counts. Plasma and serum were collected and processed as described previously (13). Shortly: PMN were isolated by Dextran-Ficoll density centrifugation (13) (purity > 95%).

### PMNs Analysis

**FACS:** PMNs (5 × 10^5^) were suspended in FACS-buffer (PBS, 10% FBS, 0.1% NaN_3_) and stained for 30 min at RT using CD66B-FITC (Biolegend), CD34-PE (Caltag) and CD61-AF647 (Biolegend). Acquisition: Accuri-C6-Plus (Becton Dickinson). PMN (10,000 events) were analyzed using Flow Jo (version 10).

**Late apoptosis**: isolated neutrophils were incubated in 100 μl annexin binding buffer containing phycoerythrin-conjugated Annexin V (Annexin V-PE) and 7-amino-actinomycin D (7-AAD) (Annexin-V Apoptosis Detection Kit, ThermoFisher Scientific) for 15 min at room temperature (RT) in the dark. After incubation, 400 μl annexin binding buffer was added, and samples were measured immediately on a BD Accuri™ C6 FACS (BD Biosciences). The Annexin V-PE^+^/7-AAD^+^ populations were taken as measurements of late apoptotic cells. Data were analyzed using FlowJo v10 software (FlowJo, LLC).

**NETs quantification**: PMN (2.5 × 10^4^) were incubated with 0.2 μM SytoxGreen (Life Technologies), 37°C, 5% CO_2_, 3 h (13). PMA (25 nM) was used as the positive control. Results are displayed as mean fluorescence intensity (MFI) measured using a Synergy H1-Hybrid-Reader (Biotek). Excitation: 485 nm/emission: 535 nm.

### Serum Analysis

**Histone/DNA complexes** was performed using the “Human Cell Death Detection ELISA^PLUS^” (Roche Diagnostics) following the manufacturing protocol (14). NE concentration was performed using the “Elastase/a1-PI Complex ELISA-Kit” (Calbiochem) following the manufacturer protocol (14).

**MPO** concentration was measured using the “Hycult Biotech ELISA-Kit” following the manufacturer protocol (14).

**CitH3** concentration was measured using “Citrullinated Histone H3 (Clone 11D3) ELISA-Kit” (Cayman) following the manufacturer protocol.

**NE activity** was measured in serum incubated with the elastase substrate, N-methoxysuccinyl-Ala-Ala-Pro-Val-7-amido-4-methylcoumarin (0.25 mM, Sigma) for 30 min at 37°C, 5% CO_2_ in the dark. The reaction product was analyzed at 360/455 nm (15).

**PAD4 western blotting:** protein extraction using RP1 Buffer (Macherey Nagel), 1% β-Mercaptoethanol (Sigma) and purification using Nucleo Spin (Macherey Nagel). Antibodies: rabbit anti-PAD4 (Abcam ab59965; 1:1,000), human anti-HRP (Cell Signaling; 1:2,000) and SuperSignal West Pico Chemiluminescent Substrate (ThermoScientific), Mouse anti-β-Actin (Sigma-Aldrich) as a control. Protein quantification was performed using Image Lab Software (version 5.2.1).

**ROS measurement:** 3.0 × 10^4^ PMNs were incubated with 25 μM DCFDH-DA (Sigma-Aldrich). Fluorescence was recorded at 0, and 30 min using a Biotek Synergy H1 Hybrid plate Reader and expressed as relative fluorescence units (RFU).

**Statistic:** All data is represented as Mean ± SEM. Comparisons between HSC donors visits was performed using ANOVA with Tukey's post-test correction. Data processed using GraphPad Prism version 8.0 (GraphPad Software Inc.).

## Results

### NET Released Is Stable Upon G-CSF Mobilization Treatment

**Figure 1:**
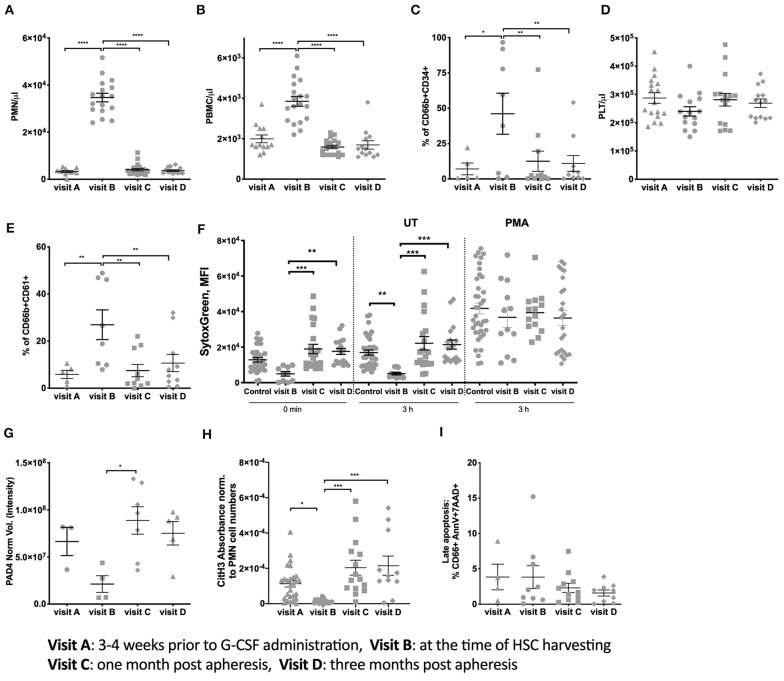
Donor PMN population characteristics and NET formation ability. **(A)** Number of PMN per μl blood of all donors. **(B)** PBMC numbers per μl blood. **(C)** Percent expression of CD66b and CD34 on isolated PMN. **(D)** Number of Platelets per μl blood of all donors. **(E)** Percent expression of CD66b and CD61 on isolated PMN. **(F)** Spontaneous and PMA induced NETosis measured using Sytox Green DNA staining of freshly isolated PMN and after 3 h incubation. This assay is performed with the same amount of cells per visit. **(G)** PAD4 expression measured from 3 × 10^6^ per visit using western blots. **(H)** CitH3 Absorbance measured in serum from donors at the different visits and normalized to PMN cell numbers. **(I)** Late apoptosis (% of total PMNs number) measured in isolated PMNs using Annexin V and 7AA-D. (^*^P < 0.05, ^**^P < 0.01, ^***^P < 0.001, ^****^P < 0.0001).

Twenty healthy stem cell donors who were undergoing HSC collection by apheresis after mobilization with G-CSF were included in the study. Demographic and clinical variables of the study population are presented in Table 1. Blood cell counting indicated a striking increase in PMN-like cells and PBMCs at the time of apheresis, which returned to control levels 1-month post-donation (Figures 1A,B). Analysis for the PMN marker CD66b and the stem cell marker CD34 indicated that a high proportion of the PMNs at the time of apheresis were of an early hemopoietic developmental stage (Figure 1C).

**Table 1:** Demographic and clinical variables of the study population.

**Blood parameters**	**Visit A ± SEM**		**Visit B ± SEM**		**Visit C ± SEM**	
Hb g/l	150	2.6	139.7	2.19	137.3	7.78
Tc G/l	277	14	240.7	15.19	278.4	20.71
Creatinine μmol/l	75	2.7	80.6	4.06		
ASAT U/l	27	1.8	32.9	2.43		
ALAT U/l	28	4.1	28.8	6.56		
CRP mg/l	4.6	1.5	17.0	3.32		
LDH U/l	209	5.6	518.8	33.9		
CD34/μl			99.9	11.89		

The formation of PMNs and platelet complexes is an essential parameter indicating the possible initiation of clotting. We observed that the number of platelets (Figure 1D) remained constant throughout the four visits. However, the percentage of PMNs positive for the platelet marker CD61 increased at visit B but with high variability between donors (Figure 1E). This increase reverted to baseline levels at later visits indicating a normalization between PMNs and platelet interactions.

Due to the expression of CD61 on PMNs the aggregation between platelets and PMNs as described in Zarbock et al. (16), could prime PMN toward NET formation. Thus, PMN NETosis was measured by staining with Sytox. At visit B, NET formation was lower compared to the baseline and reached baseline levels at visit C and D (Figure 1F). This suggests that circulating PMNs at time of HSC donation after G-CSF exposure undergo less spontaneous NETosis than at any other time points. No differences between the different groups in Sytox intensity were measured if the cells were stimulated in presence of PMA (Figure 1F).

A key feature of pro-NETotic PMNs priming is the of citrullination of histone 3 (CitH3) mediated by PAD4. A slightly lower PAD4 expression was observed at visit B compared to visit C and D (Figure 1G). Similarly, the expression of CitH3 (Figure 1H) was decreased at visit B and reached baseline levels at visit C and D. This data is in line with the decrease in spontaneous NETosis at visit B, matching the high amounts of immature PMNs. At the same time the late apoptosis of PMN, that could also be a source of free DNA in the serum, did not result in different percentage of dying cells (Figure 1I) between the analyzed time points.

### Cell-Free DNA, MPO, NE, and ROS Are Increased in Serum Upon G-CSF Mobilization Treatment

**Figure 2:**
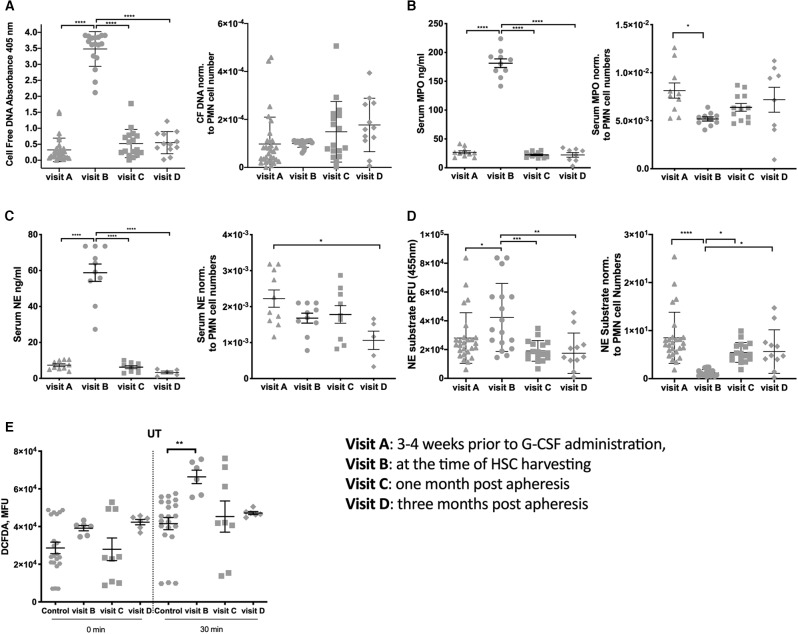
Neutrophil products are in high concentration in the blood at the time of apheresis. **(A)** Cell-free DNA in serum measured using ELISA (panel left), Cell-free DNA in serum measured using ELISA normalized data using the median per visit of PMN cell numbers (panel right). **(B)** Serum MPO concentration measured using MPO-ELISA (panel left), normalized data for serum MPO using the median per visit of PMN cell numbers (panel right). **(C)** Serum NE concentration measured using MPO-ELISA (panel left), normalized data for serum NE using the median per visit of PMN cell numbers (panel right). **(D)** NE activity measured in serum based on the ability of NE to proteolytically cleave N-methoxysuccinyl-Ala-Ala-Pro-Val-7-amido-4-methylcoumarin in order to release a fluorophore (panel left) and data normalized using the median per visit of PMN cell numbers (panel right). **(E)** Measurement of ROS release per 30,000 cells straight after plating and after 30 min incubation on a plate. (^*^P < 0.05, ^**^P < 0.01, ^***^P < 0.001, ^****^P < 0.0001).

Other factors that could induce injuries in vessels and platelet aggregation are PMN cell-free DNA, proteases MPO and NE, as previously described (17–21). Therefore, we studied the concentration of cell-free DNA, MPO and NE in the serum of donors. At visit B, a significantly higher serum concentration of cell-free DNA (Figure 2A), MPO (Figure 2B), and NE (Figure 2C) were measured compared to all other visits (all panels left). The concentration of cell-free DNA, MPO and NE were normalized to the median PMN cell number (Figures 2A–C, all panels right) to evaluate the ability of each PMN to release cell-free DNA, MPO and NE. We observed that PMNs extrude cell-free DNA, MPO, and NE at the same pace throughout the visits. Our results show that both NE activity (Figure 2D) and degranulation of MPO at visit B are decreased per cell number, reinforcing the hypothesis that probably immature circulating PMNs are not efficient in degranulation or NET formation.

As recently observed in pregnant women that G-CSF promotes PMN priming and ROS production (13), we examined the oxidative stress in our donor cohort. PMNs at visit B had a higher basal ROS secretion than controls (Figure 2E). ROS levels returned to the baseline already after 1 month. These features were more pronounced in PMN cultured for an additional 30 min, suggesting that the production of ROS is altered upon G-CSF treatment.

## Discussion

This study shows that HSC mobilization with G-CSF increases the concentration of cell-free DNA, of PMN-derived proteases and ROS in serum. This effect is transient and the measured parameters returned to baseline levels in 1-month post-treatment, suggesting the existence of a window of susceptibility to thrombotic events in predisposed subjects.

Like most growth factors and cytokines, G-CSF results in modulation of immune cell composition, cytokine profiles and immune cell response (22) in the donor. As well, in the recipient of the bone marrow transplantation, as summarized in Deotare at al. higher CD34^+^ cell doses, derived from G-CSF application, lead to better recovery of neutrophils and platelets. With related bone marrow transplant, there is no apparent association of CD34^+^ cell dose with chronic GvHD, but with unrelated bone marrow, higher CD34^+^ cell dose does seem to result in improved survival. Functional damage to the host marrow microenvironment through prior therapy and the malignant disease itself cannot be overcome by either increasing stem cell dose or using a graft with different stem cell composition (23).

Accordingly, we observe an overall increased cell number in peripheral blood of the donor, with PMN levels rising 10 times from baseline at the time of HSC collection. This increased cell number was due to the mobilization of immature PMNs as seen by the co-expression of CD66b and CD34. Although not entirely mature, about 50% of the circulating neutrophils did show a co-expression of CD61, indicating the possibility of aggregation between platelets and neutrophils. As described in Stakos et al. and Zarbock et al. this aggregation could lead to the activation of the NETotic pathway (16, 17). Another previous study proposed that the G-CSF administration could as well induce the activation of endothelial cells and the coagulation pathway, which could lead to a prothrombotic condition in stem cell donors (24). Recently Fuchs et al. (10) reported that also NETs, perfused with blood, could stimulate platelets to adhere, aggregate and promote thrombus formation. In that study, NET integrity, meaning the presence of histones on DNA-NET, was considered essential in this process because treatment with DNase and heparin (which avidly binds histones) abrogated their effect. Our study in human pregnancy did show as well a direct effect of G-CSF on the ability of PMN to release NET (13), therefore, we hypothesized that high dose G-CSF could induce spontaneous NET release, that in turn could induce thrombotic events in predisposed subjects. However, we observed that spontaneous neutrophil NET release was decreased at the time of donation and returned to control levels in 1 month after treatment. This reduced capacity to spontaneously NET formation was also mirrored by the expression of citH3 and PAD4.

Not only the spontaneous release of NET characterize the activity of PMN, but also other parameters have to be considered concerning a possible modification of the blood environment. Interestingly, we detected a 10 times higher concentration of cell-free DNA in the serum of donors after G-CSF mobilization; the origin of this cells-free DNA is still a matter of inquiring since apoptosis was not increased at visit B (data not shown). It was previously shown that increased levels of cell-free DNA in sepsis impaired fibrinolysis by inhibiting plasmin-mediated fibrin degradation (25). Another report showed that elevated levels of cell-free DNA in septic patients increased thrombin generation by activating the intrinsic pathway of blood coagulation (24). We are therefore tempted to speculate that higher cell-free DNA concentration could eventually modify the blood environment toward a predisposition to thrombotic events. However, other factors should be considered, for example the release of proteases, like, MPO and NE, which were observed in several previously published works [some examples are listed here (17–21)]. We measured also, in this case, higher concentrations of MPO and NE in serum at donation that resume control levels 1-month post-treatment.

It is important to mention that if those concentrations were normalized to the number of PMNs in the blood, the secretion of MPO and NE per cell was the same as in the other time points. This indicates that the total PMN population was not more active or prone to release DNA and proteases. The increased concentration in the serum is the consequence of high numbers of PMNs circulating in the blood and their ability to degranulate. We, therefore, propose that G-CSF creates a transient state of increased risk for hypercoagulability due to the increased concentration of cell-free DNA and proteases in the blood of HSC donors. On the other hand, thrombotic events remain infrequent, suggesting that other factors could contribute to it or prevent it.It was described that ROS could inhibit whole blood aggregation (26) and we could measure increased ROS at the time of donation. It has to be mentioned that in the work of Demers et al., performed in mice, in the presence of G-CSF, neutrophils were more sensitive to NET formation, in particular, upon encountering a “second hit,” such as low-grade infection and extracellular DNA that is generated in animals with cancer, predisposing them to thrombosis (27). This observation is extremely important, since also in our case high amount of DNA are circulating in the blood of the donor for a limited time, possibly predisposing them, in case of a secondary trigger, like an infection, to a thrombotic event.

We recognize the following limitations of the study: first of all the number of recruited patients/donors is relatively low for a comprehensive study on PMNs behavior, however, since no difference was measured in the NET formation, we are confident that the information we deliver is valuable. Second, not all samples for each time point were included in the presented analysis: for every samples we did run a technical control, if this was resulting in activated PMNs, we did excluded the donor sample from the analysis to exclude a technical error. Third, it would be important to study the interactions between all the immune cells circulating in the blood at time of G-CSF infusion, since high number of PMNs could influence the phenotype and functionality of other immune cells. It would also be interesting to investigate the correlation between neutrophils protease activity due to the increased PMNs presence in the blood and HSC mobilization from the bone marrow niches. Further investigations to better understand the pathophysiology of thromboembolic events in GCS-F mobilized donors to identify a subject at risk are needed.

## Data Availability Statement

All datasets generated for this study are included in the article.

## Ethics Statement

The studies involving human participants were reviewed and approved by Ethical committee of Northern and Central Switzerland (2015-00191). The patients/participants provided their written informed consent to participate in this study.

## Author Contributions

AH, LI, AP, and AB recruited the donor, discussed the manuscript, and organized foundings. MS, SB, GS, LV, SG, SH, and SR processed the samples, analyzed the data wrote, and discussed the manuscript.

## Conflict of Interest

The authors declare that the research was conducted in the absence of any commercial or financial relationships that could be construed as a potential conflict of interest.
